# Association between oxidative balance score and hearing loss: a cross-sectional study from the NHANES database

**DOI:** 10.3389/fnut.2024.1375545

**Published:** 2024-05-14

**Authors:** Zhongming Zhou, Yanyan Han

**Affiliations:** Department of Otolaryngology, Shanghai Punan Hospital of Pudong New District, Shanghai, China

**Keywords:** oxidative balance score, hearing loss, NHANES database, cross-sectional study, association

## Abstract

**Aim:**

The oxidative balance score (OBS), a composite score of dietary nutrients and lifestyles, reflects an individual’s oxidative and antioxidant status. Evidence showed that oxidative stress levels were related to hearing loss. The relationship between OBS and hearing loss remains unclear. This study was to explore the association between OBS and hearing loss in adults.

**Methods:**

In this cross-sectional study, data of participants aged 20–69 years who received hearing tests were extracted from the National Health and Nutrition Examination Survey (NHANES) database (2011–2012, 2015–2016). Hearing loss was defined as hearing threshold >25 dB in either ear. The OBS was composed of 16 dietary nutrients and 4 lifestyles. The covariates were screened using the backward stepwise regression analysis. The association of OBS and hearing loss was assessed with odds ratios (ORs) and 95% confidence intervals (CIs). Subgroups of age, gender, occupational noise exposure, recreational noise exposure, firearm noise exposure, and veteran status were further evaluated the associations. The importance ranking of OBS components was analyzed by the weighted random forest model.

**Results:**

Of the total 3,557 adults, 338 (9.5%) suffered from hearing loss. High OBS levels were associated with lower odds of hearing loss (OR = 0.58, 95%CI: 0.41–0.82), after adjusting age, gender, race, hypertension, tinnitus, recreational noise exposure, and occupational noise exposure. Similar results were discovered in individuals aged50-59 years old (OR = 0.47, 95%CI: 0.24–0.93), aged 60–69 years old (OR = 0.31, 95%CI: 0.16–0.61), with female (OR = 0.44, 95%CI: 0.20–0.96), without occupational noise exposure (OR = 0.31, 95%CI: 0.16–0.62), recreational noise exposure (OR = 0.48, 95%CI: 0.30–0.76), firearm noise exposure (OR = 0.38, 95%CI: 0.19–0.77), and veteran status (OR = 0.57, 95%CI: 0.39–0.82). In OBS components, vitamin B12, total fat and physical activity were important for hearing loss.

**Conclusion:**

Elevated OBS may be associated with hearing health in adults. Appropriate vitamin B12 supplementation, reduction of total fat intake, and increased physical activity may be beneficial to the prevention of hearing loss.

## Introduction

Hearing loss is a common disabling sensory dysfunction that affects approximately one in five individuals, and has become the third leading cause of years lived with disability worldwide (1, 2). The prevalence of hearing loss is rapidly increasing with the aging of the population, noise pollution, and the overuse of hearing devices (3–5). Hearing loss is usually slow-onset and associated with worse physical health, manifested as more chronic diseases and impaired physical function (6, 7). Evidence showed that people with hearing loss have difficulty to communicate with friends and family, which further limits their social networks, and may exacerbate other diseases such as depression, and dementia (6, 8, 9). Identification of indicators related to the risk of hearing loss is essential for active prevention of hearing loss and reducing the disease burden.

Aging and noise exposure are recognized as risk factors for hearing loss, and oxidative stress is one of the important causative mechanisms (10–13). Oxidative stress refers to an imbalance between reactive oxygen species (ROS) production and antioxidant defense systems (14). When there is production of ROS exceeds and insufficient endogenous processes to neutralize or detoxify them, it can lead to oxidative damage, biological membranes disruption, gene mutations, protein denaturation, and ultimately contribute to the development of various human diseases (15). Antioxidant mechanisms protect cells by resisting excess free radicals (16). Evidence showed that various dietary components such as vitamin C, vitamin E, carotenoids, and magnesium, as well as several lifestyles, such as smoking and physical activity can affect oxidative stress levels (17–19). The oxidative balance score (OBS) is a composite indicator of individual oxidative homeostasis, determined by both pro-oxidants and antioxidants, which can assess the effects of diet and lifestyle on the whole oxidative/antioxidant system (20, 21). Moreover, higher OBS reflect antioxidants exposure rather than pro-oxidation exposure and are negatively correlated with circulating oxidative stress levels (22). And OBS has proven to be an effective tool for evaluating oxidative stress status (23). Previous studies have found that high OBS levels were associated with reduced risk of several diseases, including osteoporosis, metabolic syndrome, type-2 diabetes, and breast cancer (24–27). To the beast of our knowledge, however, the association of OBS with hearing loss was unclear in adults.

This study aimed to assess the association of OBS with hearing loss, and further to explore the association in individuals with different age, gender, with or without occupational noise exposure, recreational noise exposure, firearm noise exposure, and veteran status.

## Methods

### Study design and population

All data of participants in this cross-sectional study were extracted from the National Health and Nutrition Examination Survey (NHANES) database (2011–2012 and 2015–2016), which was a program of studies to evaluate the health and nutritional status of the civilian and non-institutionalized populations in the United States. The NHANES is a multipurpose research program conducted by the National Center of Health Statistics (NCHS) and the Centers for Disease Control and Prevention (CDC) (28). The information collection was carried out through a combination of questionnaires, physical examinations, and laboratory tests (29). Additional information was available at: https://wwwn.cdc.gov/nchs/nhanes/tutorials/module2.aspx. NHANES is a publicly available dataset and was approved by the NCHS Ethics Review Board, and all patients/participants provided their written informed consent. The hospital ethics committee exempted the study from ethical review. The inclusion criteria of participants were as follows: (1) age > 20 and ≤ 69 years old; (2) without hearing-related medical conditions [ear tubes, abnormal otoscopy, impacted cerumen and abnormal tympanometry (peak pressure ≤ −150 daPa; compliance ≤0.3 mL) at either ear] (30). The exclusion criteria were: (1) missing the complete audiometric data, (2) missing the complete information for OBS assessment, (3) abnormal total energy intake levels (<500 kcal/day or > 5,000 kcal/day for female, <500 kcal/day or > 8,000 kcal/day for male), and (4) missing important co-variables [marital status, sedentary time, information of tinnitus, recreational and firearm noise exposure, body mass index (BMI)].

### OBS definition

The OBS was calculated on the basis of 4 lifestyles and 16 nutrients [4], which contains 5 pro-oxidants (smoking, alcohol consumption, BMI, iron and total fat) and 15 antioxidants (dietary fiber, selenium, copper, zinc, magnesium, calcium, calcium, vitamin E, vitamin C, vitamin B12, total folate, vitamin B6, niacin, riboflavin, and carotene). According to the OBS calculation reported by Zhang et al., the alcohol consumption was classified as non-drinkers, non-heavy drinkers (0–30 g/d for men and 0–15 g/d for women) and heavy drinkers (≥30 g/d for men and ≥ 15 g/d for women), and assigned as 2, 1 and 0 points, respectively (31). Other components were grouped first by sex and then by tertiles into 3 groups, with antioxidants scoring 0 to 2 points in groups 1 to 3 and pro-oxidants scoring 2 to 0 points in groups 1 to 3. Subsequently, factors other than alcohol consumption were grouped by sex and then by their tertiles into 3 groups, with antioxidants scoring 0 to 2 points in groups 1 to 3 and pro-oxidants scoring 2 to 0 points in groups 1 to 3 (31). Higher OBS scores indicate more significant antioxidant exposure. In this study, OBS were divided into three levels according to the tertiles, including Q1: OBS <16, Q2: 16 ≤ OBS <24, and Q3: OBS ≥24.

### Potential covariates

The potential covariates were age, gender (female and male), race (non-Hispanic White, other Hispanic, Mexican American, non-Hispanic Black, and other race-including multiracial), educational level (less than 9th grade, 9-11th grade, high school grad/general educational development (GED) or equivalent, some college or AA degree, and college graduate or above), marital status (married/living with partner, and never married/divorced/separated/widowed), poverty income ratio (PIR) (<1.0, ≥1.0, and unknown), sedentary time, height, weight, BMI, waist circumference, and total energy. Dyslipidemia was defined as triglyceride (TG) ≥150 mg/dL (1.7 mmol/L) or total cholesterol (TC) ≥200 mg/dL (5.2 mmol/L) or low-density lipoprotein cholesterol (LDL-C) ≥130 mg/dL (3.4 mmol/L) or high-density lipoprotein cholesterol (HDL-C) ≤40 mg/dL (1.0 mmol/L) or self-reported physician diagnosis or taking drug for cholesterol or taking lipid-lowering medications. Hypertension was defined as the doctor told you to have hypertension or systolic blood pressure (SBP) ≥ 130 mmHg or diastolic blood pressure (DBP) ≥ 80 mmHg, or taking blood pressure medication. Diabetes was defined as fasting glucose ≥7.0 mmol/L or glycosylated hemoglobin level (HbA1c) ≥6.5% or self-reported physician diagnosis or receiving hypoglycemic therapy. Data from the Medical Conditions Questionnaire (MCQ) were applied to identify rheumatoid arthritis; the survey question was “Has a doctor or other health professional ever told you that you had arthritis?” if your answer was yes, a follow-up question was asked “Which type of arthritis was it?” your answer was rheumatoid arthritis. Tinnitus was defined based on a response of “yes” to the question “In the past 12 months, have you been bothered by ringing, roaring, or buzzing in your ears or head that lasts for 5 min or more?” Physical activity was expressed as the metabolic equivalent task (MET) and calculated as follows: physical activity (met·min/week) = recommended MET×exercise time for corresponding activities (min/day) × the number of exercise days per week (day) (32). Carotene includes Alpha-carotene and Beta-carotene. Occupational noise exposure was evaluated with the question “Have you ever had a job, or combination of jobs where you were exposed to loud sounds or noise for 4 or more hours a day, several days a week?” (Yes/No). Firearm noise exposure was assessed by the question “Have you ever used firearms for any reason?” (Yes/No). Recreational noise exposure was evaluated by the question “Outside of a job, have you ever been exposed to very loud noise or music for 10 or more hours a week?” (Yes/No). Veteran status was evaluated as having a positive response to any of the following statements: “Have you ever served on active duty in the U.S. armed forces, military reserves, or national guard?” or “Did you ever serve in a foreign country during a time of armed conflict or on a humanitarian or peace-keeping mission?”

### Hearing loss assessment

According to the World Health Organization (WHO) standard, the average hearing threshold of 0.5, 1, 2, and 4 kHz was used to classify the severity of hearing loss in either ear as follows: mild (25–40 dB HL), moderate (40–60 dB HL), severe (60–80 dB HL), and very severe (≥80 dB HL) (33, 34). In this study, hearing loss was defined as hearing threshold >25 dB in either ear.

### Statistical analysis

The normal distribution of variables was evaluated with the Kolmogorov–Smirnov test, and the homogeneity of variables was tested with Levene’s test. Continuous data were expressed as mean ± standard deviation (mean ± SD), and the Student’s *t* test and Satterthwaite t test were used for comparison between two groups. Enumeration data were expressed as numbers and percentage [*n* (%)], and the Chi-square test was used for comparison between groups.

The covariates were screened via the step-based regression method in the weighted multivariate logistic model. Weighted univariate and multivariate logistic regression models were utilized to explore the association between OBS and hearing loss. Odds ratio (OR) and 95% confidence interval (CI) were used to assess the association. Model 1 was the univariate model. Model 2 adjusted for age, gender, race, hypertension, tinnitus, and recreational noise exposure. Model 3 adjusted for age, gender, race, hypertension, tinnitus, recreational noise exposure, and occupational noise exposure. The associations were further explored in different subgroups of age, gender, occupational noise exposure, recreational noise exposure, firearm noise exposure, and veteran status. A weighted random forest model was constructed to further explored the importance of each component in OBS.

All statistical analyses were carried out R version 4.2.3(Institute for Statistics and Mathematics, Vienna, Austria) and all data were weighted by WTMEC2YR, SDMVPSU and SDMVSTRA. *p*-value < 0.05 was considered statistically significant.

## Results

### Characteristics of participants with or without hearing loss

**Figure 1:**
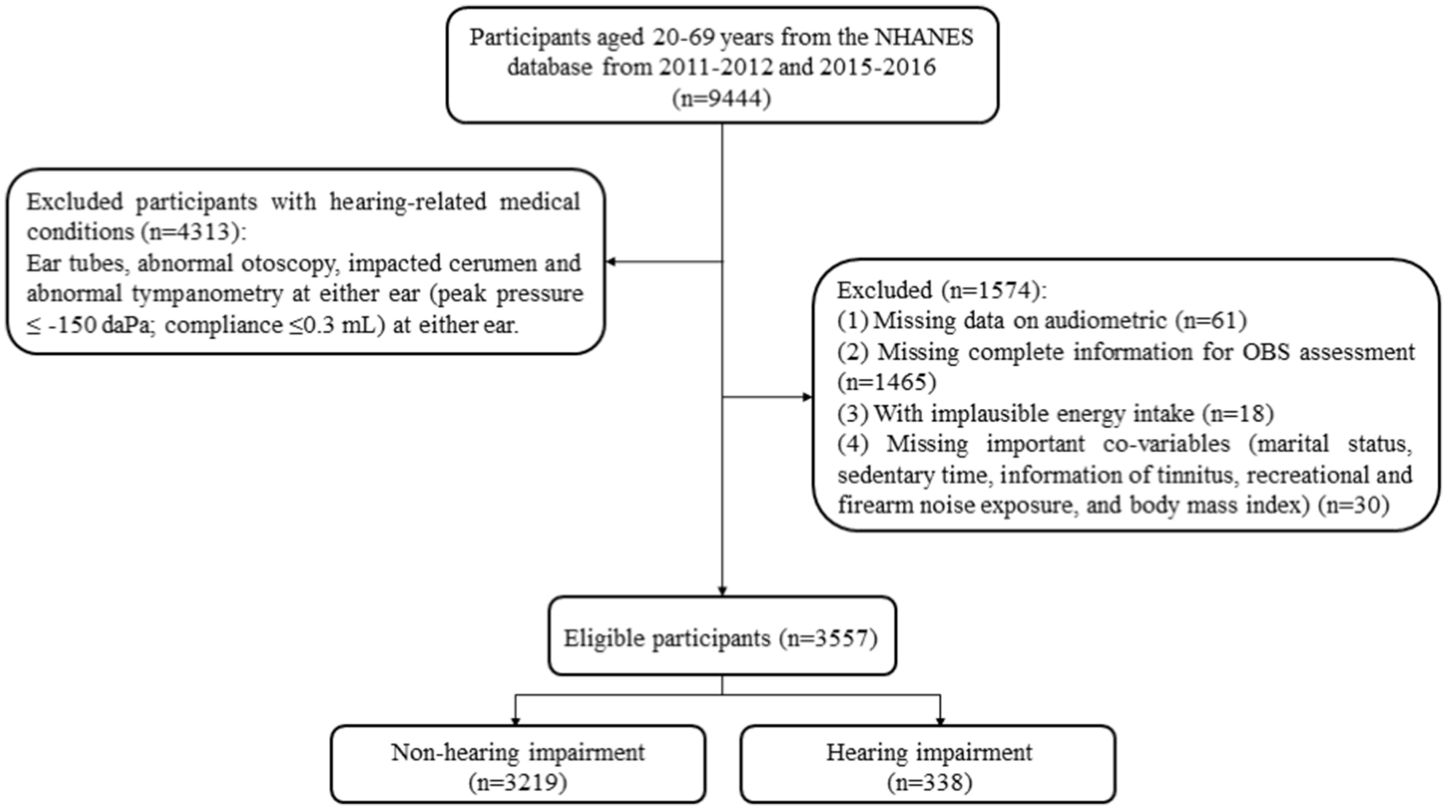
The screening flowchart of participants aged 20–69 years old.

Figure 1 shows the screening process of the participants. A total of 5,131 subjects aged 20–69 years were screened. Among them, 1,574 subjects were excluded, including 61 without the complete audiometric data, 1,465 without complete OBS assessment, 18 with abnormal total energy intake (<500 kcal/day or > 5,000 kcal/day for female, <500 kcal/day or > 8,000 kcal/day for male), and 30 without the data of marital status, sedentary time, information of tinnitus, information of recreational noise exposure, information of firearm noise exposure, and BMI. The mean age of all participants was 41.38 (13.72) years. Table 1 demonstrates the characteristics of the adults with or without hearing loss. There were statistical differences in age, gender, race, education level, hypertension, diabetes, dyslipidemia, rheumatoid arthritis, tinnitus, occupational noise exposure, recreational noise exposure, firearm noise exposure, veteran status, weight, and waist circumference (all *p* < 0.05).

**Table 1:** Characteristics of patients with hearing loss.

Variables	Total (*n* = 3,557)	Non-hearing loss (*n* = 3,219)	Hearing loss (*n* = 338)	*P*
Age, years, Mean ± SD	41.38 ± 13.72	39.96 ± 13.18	54.96 ± 11.05	<0.001^#^
Gender, *n* (%)				<0.001^*^
Female	1757 (49.4)	1,650 (51.26)	107 (31.66)	
Male	1800 (50.6)	1,569 (48.74)	231 (68.34)	
Race, *n* (%)				0.020^*^
Non-Hispanic White	1,329 (37.36)	1,192 (37.03)	137 (40.53)	
Other Hispanic	393 (11.05)	352 (10.94)	41 (12.13)	
Mexican American	496 (13.94)	438 (13.61)	58 (17.16)	
Non-Hispanic Black	793 (22.29)	740 (22.99)	53 (15.68)	
Other race-including multiracial	546 (15.35)	497 (15.44)	49 (14.5)	
Educational level, *n* (%)				<0.001^*^
Less than 9th grade	196 (5.51)	161 (5)	35 (10.36)	
9-11th grade	367 (10.32)	313 (9.72)	54 (15.98)	
High school grad/GED or equivalent	694 (19.51)	626 (19.45)	68 (20.12)	
Some college or AA degree	1,200 (33.74)	1,099 (34.14)	101 (29.88)	
College graduate or above	1,100 (30.92)	1,020 (31.69)	80 (23.67)	
Marital status, *n* (%)				0.265^*^
Married/living with partner	2,157 (60.64)	1942 (60.33)	215 (63.61)	
Never married/divorced/ separated/widowed	1,400 (39.36)	1,277 (39.67)	123 (36.39)	
PIR, *n* (%)				0.739^*^
<1.0	688 (19.34)	626 (19.45)	62 (18.34)	
≥1.0	2,623 (73.74)	2,368 (73.56)	255 (75.44)	
Unknown	246 (6.92)	225 (6.99)	21 (6.21)	
Sedentary time, min/day, Mean ± SD	364.37 ± 198.79	366.06 ± 200.31	348.34 ± 183.19	0.095^#^
Hypertension, *n* (%)				<0.001^*^
No	2037 (57.27)	1924 (59.77)	113 (33.43)	
Yes	1,520 (42.73)	1,295 (40.23)	225 (66.57)	
Diabetes, *n* (%)				<0.001^*^
No	3,132 (88.05)	2,886 (89.66)	246 (72.78)	
Yes	425 (11.95)	333 (10.34)	92 (27.22)	
Dyslipidemia, *n* (%)				<0.001^*^
No	1,280 (35.99)	1,212 (37.65)	68 (20.12)	
Yes	2,277 (64.01)	2007 (62.35)	270 (79.88)	
Rheumatoid arthritis, *n* (%)				0.003^*^
No	3,447 (96.91)	3,129 (97.2)	318 (94.08)	
Yes	110 (3.09)	90 (2.8)	20 (5.92)	
Tinnitus, *n* (%)				<0.001^*^
No	3,084 (86.7)	2,859 (88.82)	225 (66.57)	
Yes	473 (13.3)	360 (11.18)	113 (33.43)	
Occupational noise exposure, *n* (%)				<0.001^*^
No	2,330 (65.5)	2,167 (67.32)	163 (48.22)	
Yes	1,227 (34.5)	1,052 (32.68)	175 (51.78)	
Recreational noise exposure, *n* (%)				<0.001^*^
No	3,049 (85.72)	2,785 (86.52)	264 (78.11)	
Yes	508 (14.28)	434 (13.48)	74 (21.89)	
Firearm noise exposure, *n* (%)				<0.001^*^
No	2045 (57.49)	1892 (58.78)	153 (45.27)	
Yes	1,512 (42.51)	1,327 (41.22)	185 (54.73)	
Veteran status, *n* (%)				<0.001^*^
No	3,318 (93.28)	3,041 (94.47)	277 (81.95)	
Yes	239 (6.72)	178 (5.53)	61 (18.05)	
Height, cm, Mean ± SD	168.57 ± 9.88	168.48 ± 9.91	169.44 ± 9.62	0.091^&^
Weight, kg, Mean ± SD	83.22 ± 21.95	82.93 ± 22.07	85.94 ± 20.59	0.017^&^
BMI, kg/m^2^, Mean ± SD	29.21 ± 7.00	29.14 ± 7.06	29.86 ± 6.46	0.053^#^
Waist circumference, cm, Mean ± SD	98.51 ± 16.68	97.98 ± 16.66	103.49 ± 16.08	<0.001^&^
Total energy, kcal/day, Mean ± SD	2249.47 ± 957.11	2248.29 ± 958.58	2260.64 ± 944.31	0.822^&^
OBS, score, Mean ± SD	19.53 ± 7.16	19.57 ± 7.17	19.15 ± 6.99	0.303^&^
OBS, *n* (%)				0.583^*^
Q1 (<16)	1,326 (37.28)	1,197 (37.19)	129 (38.17)	
Q2 (16–24)	1,102 (30.98)	992 (30.82)	110 (32.54)	
Q3 (≥24)	1,129 (31.74)	1,030 (32)	99 (29.29)	

*Chi-square test; ^#^Satterthwaite *t* test; ^&^Student’s *t* test; SD: standard deviation. GED: general educational development; PIR: poverty income ratio; BMI: body mass index; OBS: oxidative balance score.

### Association of OBS with hearing loss in adults

The relationships between OBS levels and hearing loss in adults were shown in Table 2. Compared with OBS <16, the OBS ≥24 was associated with lower odds of hearing loss in adults (OR = 0.58, 95%CI: 0.41–0.82, *p* = 0.005), after adjustments for age, gender, race, hypertension, tinnitus, recreational noise exposure, and occupational noise exposure.

**Table 2:** Association of OBS with hearing loss in adults.

Variables	Model 1		Model 2		Model 3	
	OR (95%CI)	*P*	OR (95%CI)	*P*		
OBS						
Q1 (<16)	Ref		Ref		Ref	
Q2 (16–18)	0.98 (0.62–1.57)	0.949	0.99 (0.58–1.70)	0.970	1.00 (0.60–1.66)	0.986
Q3 (≥24)	0.62 (0.44–0.89)	0.013	0.57 (0.39–0.82)	0.005	0.58 (0.41–0.82)	0.005

OBS: ondansetron; OR: odd ratio; CI: confidence interval. Model 1: crude model. Model 2: adjusted for age, gender, race, hypertension, tinnitus, and recreational noise exposure. Model 3: adjusting for age, gender, race, hypertension, tinnitus, recreational noise exposure, and occupational noise exposure.

### Associations of OBS with hearing loss in different groups of age, gender, occupational noise exposure, recreational noise exposure, firearm noise exposure, and veteran status

**Figure 2:**
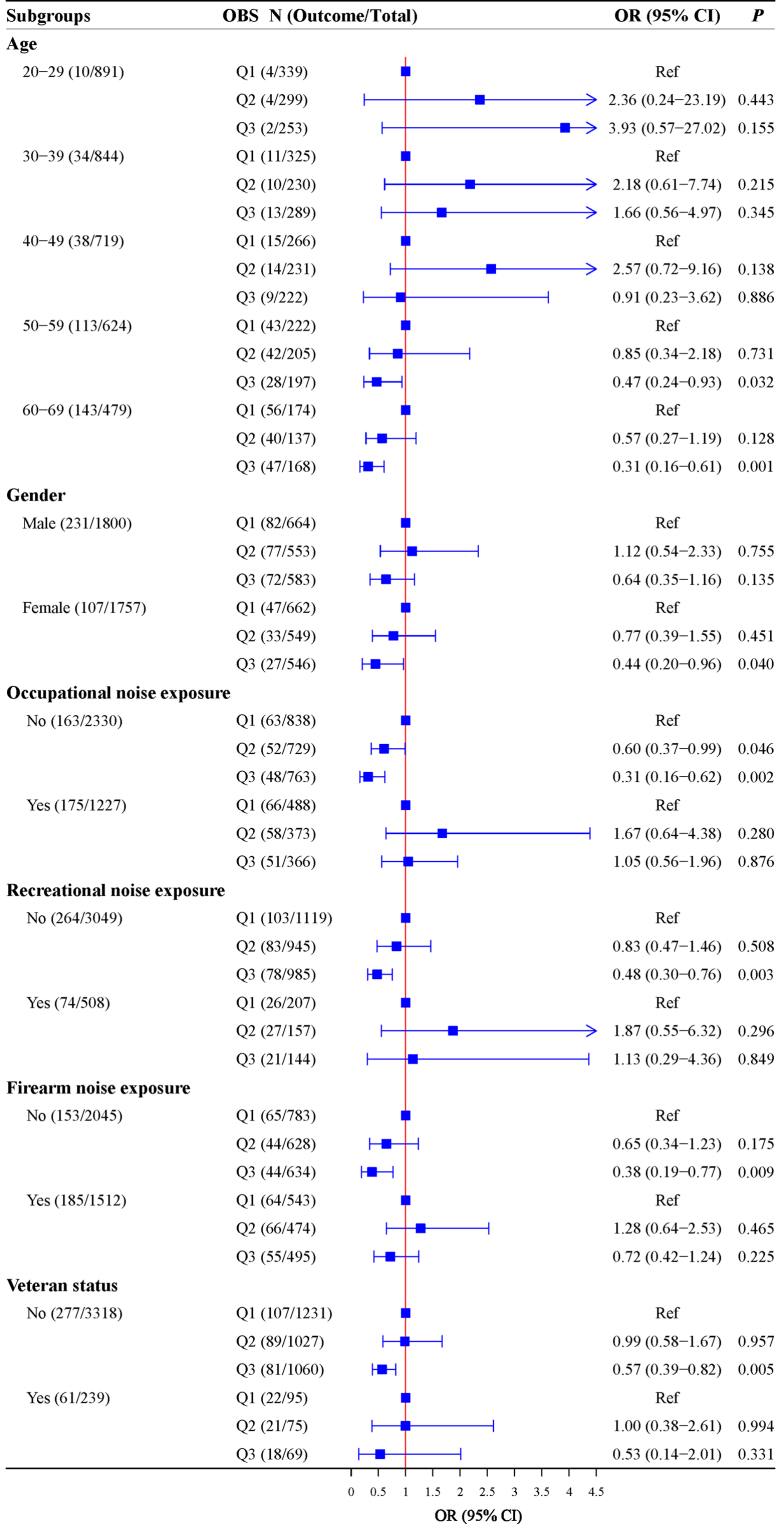
Associations of OBS with hearing in subgroups of age, gender, occupational noise exposure, recreational noise exposure, firearm noise exposure, and veteran status.

Further analyses were conducted to explore the relationship between OBS and hearing loss in different age, gender, occupational noise exposure, recreational noise exposure, firearm noise exposure, and veteran status groups. High OBS levels were associated with lower odds of hearing loss in participants aged 50–59 years old (OR = 0.47, 95%CI: 0.24–0.93), aged 60–69 years old (OR = 0.31, 95%CI: 0.16–0.61), with female (OR = 0.44, 95%CI: 0.20–0.96), without occupational noise exposure [Q2: (OR = 0.60, 95%CI: 0.37–0.99); Q3: (OR = 0.31, 95%CI: 0.16–0.62)], without recreational noise exposure (OR = 0.48, 95%CI: 0.30–0.76), without firearm noise exposure (OR = 0.38, 95%CI: 0.19–0.77), and without veteran status (OR = 0.57, 95%CI: 0.39–0.82). Detailed results are shown in Figure 2.

### The importance ranking of OBS components

**Figure 3:**
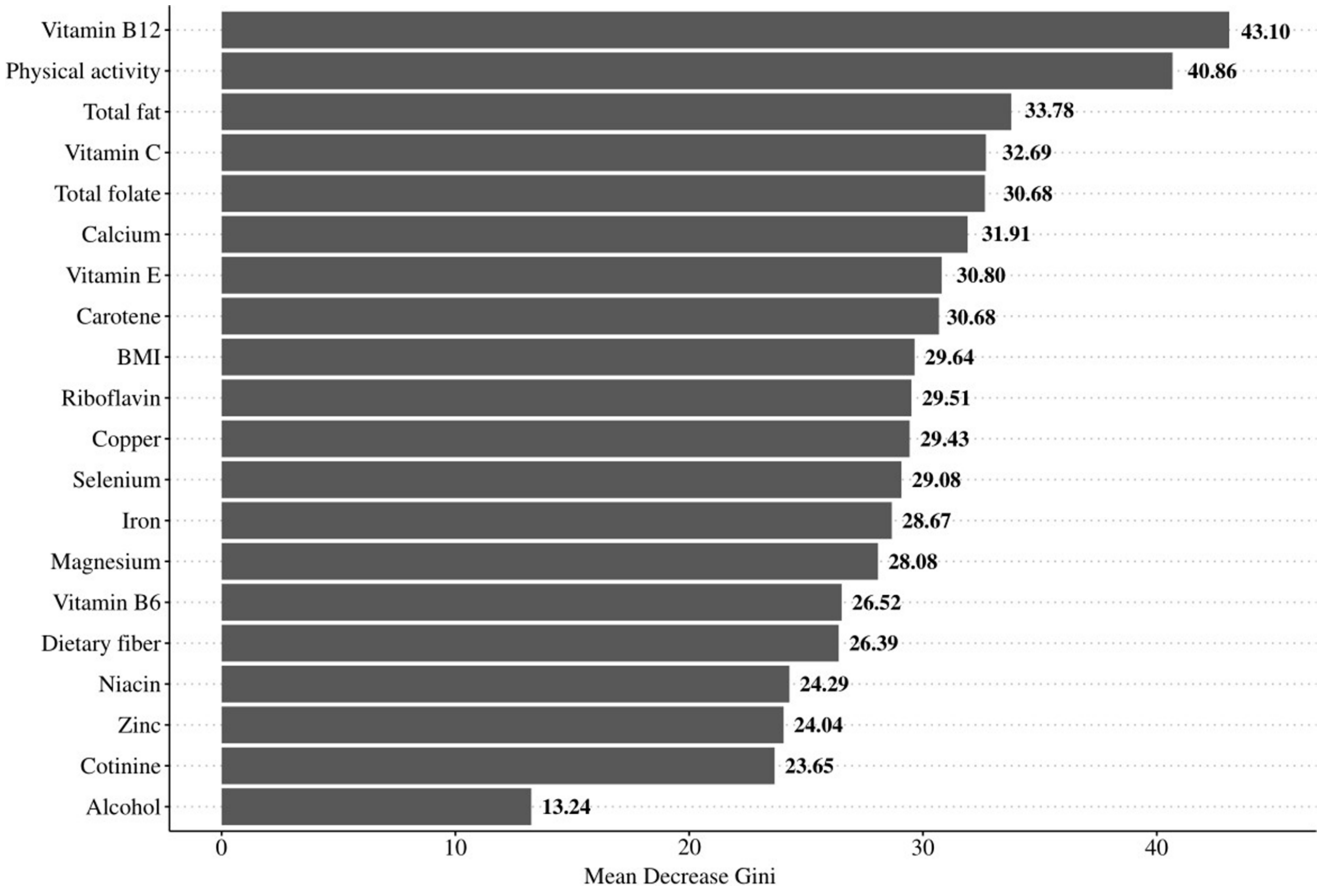
The importance ranking of OBS components.

The weighted random forest model was used to further examine the importance of each component in OBS. Figure 3 shows that the Mean Decrease Gini of vitamin B12, total fat and physical activity were 43.10, 40.68 and 33.78, respectively, suggesting that vitamin B12, total fat and physical activity were important for hearing loss.

## Discussion

This study aimed to investigate the effect of OBS on hearing loss in U.S. adults. Our findings showed that high OBS levels were associated with lower odds of hearing loss. Similar results were discovered in patients aged 50–69 years old, female, without occupational noise exposure, recreational noise exposure, firearm noise exposure, and veteran status. In OBS components, vitamin B12, total fat and physical activity were important for hearing loss.

Hearing loss is a slow-onset and progressively worsening sensory dysfunction (35). Oxidative stress, as one of the most extensively studied factors contributing to hearing loss, is due to an imbalance between ROS production and antioxidant defense systems (36). Evidence showed that various dietary components and lifestyles may affect the oxidative stress levels in the body, and may be associated with the risk of hearing loss (17–19, 37). Choi et al. (19) found that high vitamin C, vitamin E and magnesium were negatively correlated with the risk of hearing loss, and there was a synergistic effect between different nutrients, that is, combined intake of β-carotene, vitamin C and magnesium may contribute to lower hearing thresholds. Jung et al. (38) found that higher magnesium intake was associated with decreased risk of hearing loss. Kawakami et al. (18) reported that vigorous-intensity leisure-time physical activity was associated with decreased risk of hearing loss. A single factor has a limited effect on the whole oxidation/antioxidant system, while the combined effect of multiple factors may be more closely related to health outcomes (39, 40). The OBS serves as a comprehensive measure of individual oxidative homeostasis, reflecting the balance between pro-oxidants and antioxidants, and can be utilized to assess diseases associated with oxidative stress exposure (24–27, 41). Golmohammadi et al. (24) found that higher OBS, indicating more antioxidants exposure than pro-oxidants exposure, was associated with better glycemic control in Iranian adults with type-2 diabetes. Liu et al. (41) reported that higher OBS, that is, more antioxidants than pro-oxidants exposure in dietary components and lifestyles was associated with lower incidence of depression. Herein, we found that high OBS was associated with lower odds of hearing loss in adults. The vitamin B12, total fat and physical activity were important in OBS components for hearing loss. Abbasi et al. (42) reported that vitamin B12 may have a protective effect as an antioxidant on occupational hearing loss. Kim et al. (43) reported that low fat intakes were associated with hearing discomfort among the elderly of Korea. Kawakami et al. (44) found that higher muscular and performance fitness were associated with decreased risk of hearing loss.

We further evaluated the association of OBS with hearing loss in different populations. Our results showed that high OBS was associated with lower odds of hearing loss in adults aged 50–69 years old, with female, without occupational, recreational and firearm noise exposure, and veteran status. The high OBS seemed to not be associated with a risk of hearing loss in individuals aged <50 years old, who were regularly exposed to occupational, recreational and firearm noise exposure, and with veteran status, which may be related to excessive recreational, occupational and firearm noise exposures in this population (45–47). Evidence suggested that an increase in ROS, reactive nitrogen species, and lipid peroxides after chronic occupational noise exposure, causing oxidative stress (48), which was no association between OBS and hearing loss risk. Our study found that high OBS seemed to not be associated with hearing loss in males, one reason may be that men smoke more intensely than women and are more likely to be affected by the oxidative stress caused by smoking and the oxidative burden that remains even after quitting compared with never smoking (17, 49). In addition, men are generally exposed to more noise exposure than women (45).

Several possible mechanisms may explain the relationship between high OBS and lower odds of hearing loss. The higher OBS reflect antioxidants exposure rather than pro-oxidation exposure and are negatively correlated with circulating oxidative stress levels (22). The formation of ROS such as superoxide anions, hydrogen peroxide, and hydroxyl radicals is a key mechanism of hearing loss, leading to inner ear hair cell death and consequence vasoconstriction and a rebound of cochlear blood flow (48, 50, 51). Antioxidants have powerful ROS scavenging activity due to their unique membrane function and ability to penetrate the blood–brain barrier (52). Animal experiments have shown that antioxidants such as β-carotene and/or vitamins C and/or E and/or magnesium play a role in the prevention or treatment of hearing loss by reducing noise-induced free radical formation, and inner ear hair cell death (53–55).

OBS is a composite indicator that can assess diet and lifestyle exposures related to oxidative stress. The present study found an association between high OBS and the lower odds of hearing loss in adults. The findings of our study have potential implications for the clinical management and prevention of hearing loss. Appropriate vitamin B12 supplementation, reduction of total fat intake, and increased physical activity are beneficial to the prevention of hearing loss. Future studies are needed to confirm the association between OBS and hearing loss in adults.

The current study also has several limitations. First, this study was a cross-sectional study, it is difficult to establish a causal association between OBS and hearing loss. Second, potential covariates such as noise exposure were considered in this study, but possible influencing factors such as hearing protection measures in noise environments were not available.

## Conclusion

High OBS levels were associated with lower odds of hearing loss in U.S. adults, especially in individuals aged ≥45 years old, with female, without occupational, recreational and firearm noise exposure, and veteran status. Appropriate vitamin B12 supplementation, reduction of total fat intake, and increased physical activity are beneficial to the prevention of hearing loss.

## Data availability statement

Publicly available datasets were analyzed in this study. This data can be found at: NHANES database, https://wwwn.cdc.gov/nchs/nhanes/.

## Ethics statement

The requirement of ethical approval was waived by Shanghai Punan Hospital of Pudong New District for the studies involving humans because the data was accessed from a publicly available database. The studies were conducted in accordance with the local legislation and institutional requirements. The participants provided their written informed consent to participate in this study.

## Author contributions

ZZ: Conceptualization, Data curation, Formal analysis, Investigation, Methodology, Project administration, Supervision, Writing – original draft, Writing – review & editing. YH: Conceptualization, Formal analysis, Funding acquisition, Project administration, Supervision, Writing – review & editing.
