# Supplementary material for: Association between oxidative balance score and hearing loss: a cross-sectional study from the NHANES database
Source: Front Nutr. 2024 May 14;11:1375545. doi: 10.3389/fnut.2024.1375545 (PMC11135173; doi:10.3389/fnut.2024.1375545)
Supplement: Supplementary file 1 [file Table_1.DOCX]

Table S1 Screening for potential covariates of hearing loss risk

| Variables | Model 1 | | Model 2 | |
| --- | --- | --- | --- | --- |
|  | OR (95% CI) | *P* | OR (95% CI) | *P* |
| Age | 1.10 (1.08-1.12) | <0.001 | 1.10 (1.09-1.12) | <0.001 |
| Gender |  |  |  |  |
| Female | Ref |  | Ref |  |
| Male | 2.14 (1.46-3.13) | <0.001 | 2.33 (1.49-3.64) | 0.001 |
| Race |  |  |  |  |
| Non-Hispanic White | Ref |  | Ref |  |
| Other Hispanic | 0.55 (0.35-0.88) | 0.020 | 0.94 (0.54-1.66) | 0.837 |
| Mexican American | 0.64 (0.41-0.99) | 0.055 | 1.33 (0.78-2.27) | 0.278 |
| Non-Hispanic Black | 0.49 (0.34-0.72) | 0.001 | 0.62 (0.41-0.95) | 0.029 |
| Other Race-Including Multi-Racial | 1.01 (0.61-1.66) | 0.976 | 1.45 (0.85-2.46) | 0.161 |
| Educational level |  |  |  |  |
| Less than 9th grade | Ref |  |  |  |
| 9-11th grade | 1.18 (0.62-2.25) | 0.614 |  |  |
| High school grad/GED or equivalent | 0.99 (0.51-1.93) | 0.976 |  |  |
| Some college or AA degree | 0.66 (0.34-1.27) | 0.221 |  |  |
| College graduate or above | 0.62 (0.31-1.24) | 0.185 |  |  |
| Marital status |  |  |  |  |
| Married/living with partner | Ref |  |  |  |
| Never married/divorced/ separated/widowed | 0.90 (0.61-1.34) | 0.613 |  |  |
| PIR |  |  |  |  |
| <1.0 | Ref |  |  |  |
| ≥1.0 | 1.72 (0.99-3.00) | 0.064 |  |  |
| Unknown | 1.24 (0.49-3.13) | 0.658 |  |  |
| Sedentary time | 1.00 (1.00-1.00) | 0.382 |  |  |
| Hypertension |  |  |  |  |
| No | Ref |  | Ref |  |
| Yes | 3.20 (2.37-4.33) | <0.001 | 1.48 (1.00-2.20) | 0.050 |
| Diabetes |  |  |  |  |
| No | Ref |  |  |  |
| Yes | 2.87 (1.76-4.69) | <0.001 |  |  |
| Dyslipidemia |  |  |  |  |
| No | Ref |  |  |  |
| Yes | 2.12 (1.44-3.13) | 0.001 |  |  |
| Rheumatoid arthritis |  |  |  |  |
| No | Ref |  |  |  |
| Yes | 2.78 (1.12-6.90) | 0.035 |  |  |
| Tinnitus |  |  |  |  |
| No | Ref |  | Ref |  |
| Yes | 4.17 (2.88-6.04) | <0.001 | 2.59 (1.64-4.10) | <0.001 |
| Occupational noise exposure |  |  |  |  |
| No | Ref |  |  |  |
| Yes | 2.02 (1.45-2.83) | <0.001 |  |  |
| Recreational noise exposure |  |  |  |  |
| No | Ref |  | Ref |  |
| Yes | 1.93 (1.23-3.01) | 0.007 | 2.06 (1.21-3.50) | 0.010 |
| Firearm noise exposure |  |  |  |  |
| No | Ref |  |  |  |
| Yes | 1.56 (1.05-2.34) | 0.037 |  |  |
| Veteran status |  |  |  |  |
| No | Ref |  |  |  |
| Yes | 2.70 (1.63-4.47) | 0.001 |  |  |
| Total energy | 1.00 (1.00-1.00) | 0.426 |  |  |

Ref: reference, OR: odd ratio, CI: confidence interval.

GED: general educational development; PIR: poverty income ratio.

Model 1: the weighted univariate logistic regression model.

Model 2: the weighted multivariate logistic regression model.
